# Midterm outcome after posterior stabilization of unstable Midthoracic spine fractures in the elderly

**DOI:** 10.1186/s12891-021-04049-3

**Published:** 2021-02-15

**Authors:** U. J. Spiegl, P.-L. Hölbing, J.-S. Jarvers, N. v. d. Höh, P. Pieroh, G. Osterhoff, C.-E. Heyde

**Affiliations:** Department of Orthopaedics, Trauma Surgery and Plastic Surgery, University of Leipzig, Liebigstr. 20, 04103 Leipzig, Germany

**Keywords:** Osteoporotic vertebral body fracture, Midthoracic spine, Posterior stabilization, Long segmental posterior stabilization, Thoracic cage injury

## Abstract

**Background:**

The evidence for the treatment of midthoracic fractures in elderly patients is weak. The aim of this study was to evaluate midterm results after posterior stabilization of unstable midthoracic fractures in the elderly.

**Methods:**

Retrospectively, all patients aged ≥65 suffering from an acute unstable midthoracic fracture treated with posterior stabilization were included. Trauma mechanism, ASA score, concomitant injuries, ODI score and radiographic loss of reduction were evaluated. Posterior stabilization strategy was divided into short-segmental stabilization and long-segmental stabilization.

**Results:**

Fifty-nine patients (76.9 ± 6.3 years; 51% female) were included. The fracture was caused by a low-energy trauma mechanism in 22 patients (35.6%). Twenty-one patients died during the follow-up period (35.6%). Remaining patients (*n* = 38) were followed up after a mean of 60 months. Patients who died were significantly older (*p* = 0.01) and had significantly higher ASA scores (*p* = 0.02). Adjacent thoracic cage fractures had no effect on mortality or outcome scores. A total of 12 sequential vertebral fractures occurred (35.3%). The mean ODI at the latest follow up was 31.3 ± 24.7, the mean regional sagittal loss of reduction was 5.1° (± 4.0). Patients treated with long segmental stabilization had a significantly lower rate of sequential vertebral fractures during follow-up (*p* = 0.03).

**Conclusion:**

Unstable fractures of the midthoracic spine are associated with high rates of thoracic cage injuries. The mortality rate was rather high. The majority of the survivors had minimal to moderate disabilities. Thereby, patients treated with long segmental stabilization had a significantly lower rate of sequential vertebral body fractures during follow-up.

## Introduction

Operative reduction and stabilization is indicated in unstable vertebral body fractures of the thoracolumbar spine. Several therapy strategies have been reported in the treatment of these fractures in the elderly, ranging from cement augmented procedures such as kyphoplasty or vertebroplasty, short and long segmental stabilizations and hybrid stabilizations [1–6]. The majority of those studies focuses on the thoracolumbar junction and the lumbar spine [2, 4–6]. However, the anatomy and biomechanics of the kyphotic mid-thoracic spine differ tremendously from the thoracolumbar junction, and the lordotic lumbar spine. The vertebral bodies including their pedicles are smaller at the thoracic spine [7, 8]. The thoracic cage leads to a higher stiffness [9]. The kyphotic alignment causes higher axial loads at the anterior part of the vertebral bodies in standing position [10]. Additionally, those fractures are associated with a high rate of concomitant injuries of the thoracic cage, which might influence the outcome negatively, particularly in a geriatric patient population. Thus, the comparability regarding treatment strategies and outcome between midthoracic fractures and fractures of the thoracolumbar junction and the lumbar spine is questionable. To the best of the authors’ knowledge there exist no studies dealing specifically with unstable midthoracic fractures in the elderly treated with posterior stabilization.

The aim of this study was to evaluate the clinical and radiographic midterm results of posterior stabilization for the treatment of unstable fractures of the midthoracic spine in patients aged 65 years or older. The first hypothesis was that midthoracic fractures are often caused by low and moderate energy trauma and are associated with a high rate of adjacent injuries of the thoracic cage which leads to inferior outcomes. The second hypothesis states that posterior stabilization leads to mainly good clinical and radiographic outcome. The third hypothesis was that patients might benefit from a long segmental stabilization (LSS).

## Methods

The study was performed at a single level I trauma center between January 2010 and December 2017. The patient enrollment was done retrospectively; the patients were examined at the follow-up prospectively. The study was approved by the institutional ethics committee. All patients admitted with spinal injury were examined clinically and received conventional radiographs after low or moderate energy trauma and a whole body computed tomography (CT) after high energy accidents. A magnetic resonance imaging (MRI) of the whole spine was performed in those patients without MRI contraindications. Additionally, CT was carried out in patients with signs of vertebral fractures after low and moderate energy traumas with contraindication for MRI, and patients suffering from ankylosing spondylitis or patients with signs of concomitant injuries. All concomitant injuries were analyzed, particularly those of the thoracic cage. The trauma mechanism was analyzed and divided into non memorable, low energy trauma, moderate energy trauma, and high energy trauma. Low energy trauma was defined as stumbling while walking or falling while standing. Moderate energy trauma was defined as traffic accidents with low velocity (≤ 30 km/h) and falls above standing height to 3 m, whereas high energy trauma was defined as falls from height of greater than 3 m and car accidents with higher velocities (> 30 km/h).

Vertebral body fractures were classified in accordance to the OF-classification [11]. OF type 5 fractures were additionally classified in accordance to the AO spine classification [12]. All patients underwent a thorough neurological examination using the ASIA protocol [13]. Patients with neurologic deficits were excluded. Conventional radiographs in standing position were performed before mobilization as well as after mobilization. Unstable fractures were defined by an OF type 4 and 5 as well as OF type 3 fractures with a bisegmental reduction of more than 5° after mobilization. Generally, the indication for surgery was seen in accordance to Blattert et al. [14] in patients with an OF type 6 and higher (Table 1). Inclusion and exclusion criteria are listed in Table 2. Prior to surgery the ASA (American Society of Anesthesiologists) score was evaluated in all patients [15] and the presence of following risk factors was recorded based on chart review: Diabetes mellitus, cardiac insufficiency, renal insufficiency, and chronic obstructive pulmonary disease (COPD).

**Table 1:** Definition of the OF-Score in accordance to Blattert et al. [14]

OF-Score		
Parameter	Grade	Points
Fracture classification type (OF 1–5) [11]	1–5	2–10
Bone mineral density	T-score < − 3	1
Ongoing fracture process	Yes; No	1; −1
Pain (under analgesia)	VAS ≥4; < 4	1; −1
Neurological deficit	Yes	2
Mobilization (under analgesia)	No; Yes	1; − 1
Health status	ASA > 3; dementia; BMI > 20 kg/m^2^; nursing case; anticoagulation	Each −1; Max. -2

Abbreviations: *ASA* American Society of Anesthesiologists risk classification, *BMI* Body mass index, *VAS* Visual analogue scale for pain, *Max*. Maximum

0–5 points: nonsurgical; 6 points: nonsurgical or surgical; > 6 points: surgical

**Table 2:** Inclusion and Exclusion Criteria

Inclusion Criteria	Exclusion Criteria
Age: ≥ 65 years	Prior or subsequent fractures of the vertebral spine caused by another trauma
OF-score ≥ 6 [14]	Inability or unwillingness to join the study
Location: Th3 – Th10	Neurologic impairment
Posterior stabilization	Pathologic vertebral body fractures (tumor/infection)
Acute fracture situation	Conservative treatment

*Th* Thoracic vertebral body

### Surgical techniques

Posterior stabilization was done via a minimally invasive or open midline approach. Posterior stabilization was done with cement-augmented pedicle screws (Matrix, Fa. DepuySynthes; Viper, Fa. DepuySynthes; Longitude, Fa. Medtronic,; MUST, Fa. Medacta). The approach and the implant were chosen as preferred by the surgeon. A total of seven experienced spine surgeons performed the surgeries. All patients with adjacent fractures of the thoracic cage were treated with LSS. All others were treated either with LSS (≥ 4 segments) or with short segmental stabilization (SSS) (≤ 3 segments).

### Postoperative management

All patients received conventional standing radiographs. An additional CT scan was performed in symptomatic patients and in patients, in whom the implant position could not be evaluated sufficiently by conventional radiographs. No braces or corsets were subscribed. Physiotherapy was started the day after surgery. Clinical and radiological follow-up was performed after 2, 6, 12 weeks, and 1 year after surgery. Dual X-Ray Absorptiometry (DXA) assessment and an anti-osteoporotic therapy were recommended to all patients.

### Outcome parameters

All patients were followed for at least 18 months after initial surgery. All patients were initially called by phone and asked if they were willing to participate. Thereby, all patients were asked about their current anti-osteoporotic therapy. Patients were invited for clinical and radiological evaluation and were asked to complete clinical scores. Anterior-posterior conventional radiographs centered on the injured vertebral body were performed including lateral 36 in. views in standing position. Clinical scores were mailed to those patients who could not attend the follow-up examination.

### Outcome measures

**Fig. 1:**
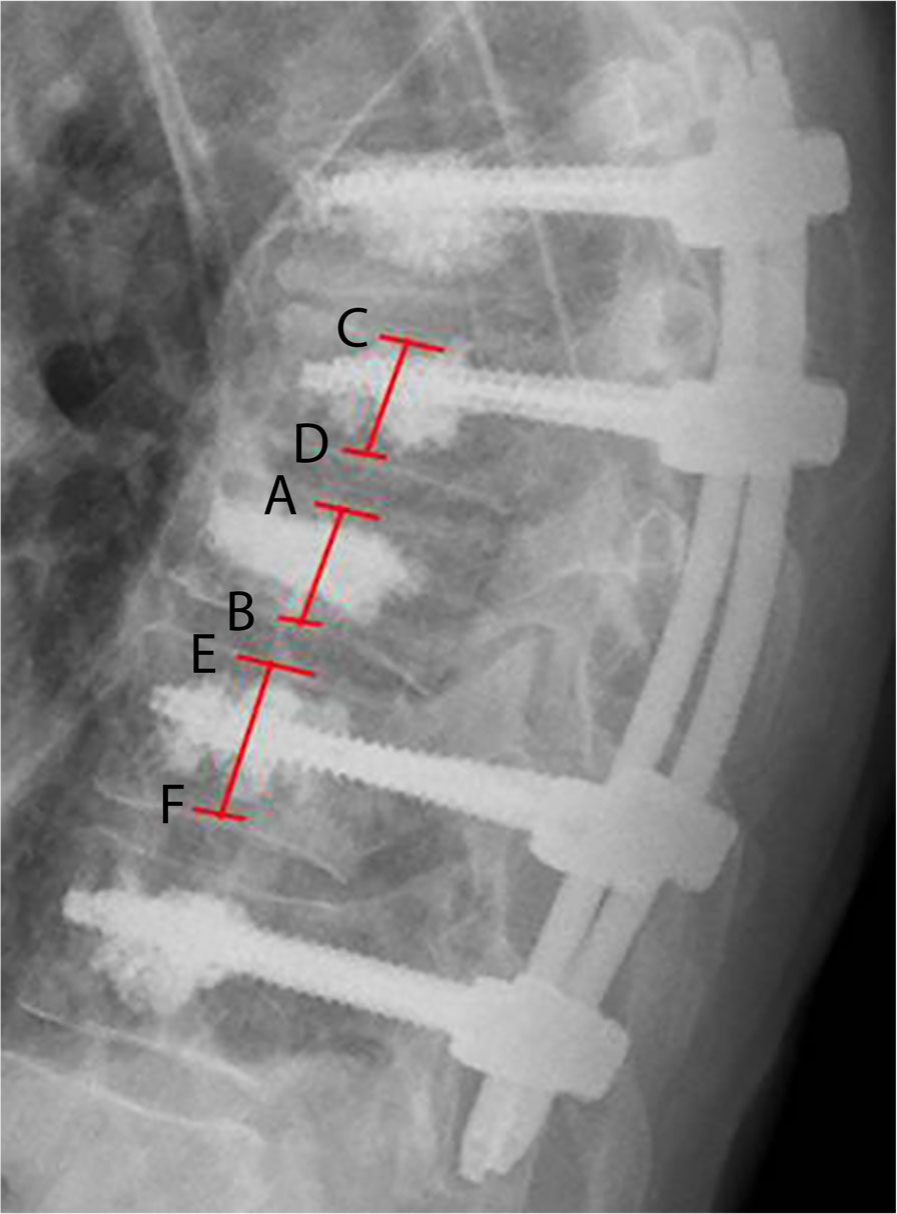
Depicted is the definition of the relative medial vertebral body height: (AB / $\frac{CD+ EF}{2}$)*100 in percent. Thereby the height is measured at midpoint of the vertebral body in the sagittal view

The clinical primary parameter of interest was the Oswestry Disability Index (ODI) at the last follow-up. The radiological primary parameter was the radiologic loss of reduction (bisegmental Cobb angle) [16]. Further outcome parameters were the pain level (VAS 0–10 scale; 0: no pain, 10: maximal pain), the satisfaction level (VAS 0–10 scale; 0: lowest satisfaction, 10: highest), the SF-36 score (physical summary component and mental summary component) [17], the Timed-Up-and-Go test [18], the complication rates, and surgical revisions. Additional radiological parameters were the following: relative medial vertebral body height (Fig. 1), pelvic tilt, pelvic incidence, sacral slope, lumbar lordosis, thoracic kyphosis, C7 plumb line, signs of instability, and signs of implant loosening. Additionally, the rate of further sequential vertebral fractures was evaluated.

### Statistics

Statistical analyses were performed with standardized SPSS software 24.0 (SPSS®, Inc. Chicago, USA using descriptive statistics. Two-sample Wilcoxon signed-rank tests were employed to compare outcome parameters comparing differences between LSS and SSS. Fisher’s exact test and Pearson test were used to evaluate any correlations between clinical and radiological outcome parameters, and potential risk factors, the injury pattern including all adjacent injuries and the clinical outcome, and between regional radiological outcome parameters and alignment parameters. A significance level of 0.05 was used.

## Results

A total of 59 patients met the inclusion criteria (Table 2). The average age was 76.9 years (range 65 to 89 years). The rate of males and females was equal (49% vs. 51%). The trauma mechanism could not be remembered in 13 cases (22.0%). Twenty-one patients (35.6%) had a low energy trauma, 13 patients (22.0%) experienced a moderate energy trauma, whereas twelve patients (20.3%) suffered from a high energy trauma. There was a significant correlation between the trauma mechanism and concomitant thoracic injuries (*p* < 0.001). The majority of patients who suffered from a high energy trauma had concomitant thoracic injuries (83.3%). The rate of concomitant thoracic injuries of patients with moderate, low, or nonmemorable energy trauma mechanisms was 30.8, 4.8, and 7.7% respectively. The average follow-up was 59.9 months (median: 56.6 months; range: 18–111 months). A total of 21 patients (mean age at the time of surgery: 79.4 years) died during the follow-up period (35.6%). One of those patients died during post-operative hospital stay from a pulmonary embolism. Four further patients were lost to follow-up (mean age: 75.0 years; range: 65–83) whereas 34 of the surviving 38 patients could be re-evaluated (89.5%). The average age at surgery of the patients who could be re-evaluated was 75.4 years (range 66–84 years). The genders in this group were equally distributed (*n* = 17/17). Patients who died during the follow-up period were significantly older (*p* = 0.014) and had higher ASA scores at the time of surgery (non-survivors: 2.7 vs survivors: 2.4; *p* = 0.022). There were no further statistically significant differences between survivors and non-survivors with respect to fracture location, fracture classification, trauma mechanism, treatment strategy, adjacent injuries, as well as surgical approach, and time of surgery (Table 3). The majority of fractures of the patients that were re-evaluated occurred at the thoracic (Th) levels 7, 8, and 9 (*n* = 21; 62%). Most fractures were complete burst fractures (18x OF type 4; 53%), less frequently incomplete burst fractures of type OF 3 (*n* = 6: 18%) or unstable OF 5 fractures (*n* = 10; 29%). Ten patients suffered from concomitant fractures of the thoracic cage (29.4%) consisting of unilateral rib series fractures in seven patients and bilateral rib series fractures in two patients, all of whom had some degree of lung parenchyma injuries. Thereby, chest tubes were placed in four patients. None of the thoracic cage injuries were treated operatively. Fractures of the sternum were seen in two patients and two patients suffered from clavicle fractures. Osteosynthesis with a plate was performed in one of the patients suffering from a clavicle fracture. The mean ODI at the latest follow up was 31.3% (range: 0–80%). Thereby, 14 patients (41.1%) had a minor disability, 17.6% a moderate disability, 32.3% a severe disability, and three patients (8.8%) crippling back pain. Two of the three patients with the highest ODI-scores had sequential vertebral fractures and the third patient was 90 years old and frail at last follow-up. The mean radiologic loss of reduction was 5.1° (range: 1° – − 11°). At the final follow-up the medial vertebral body height was 70.3% (± 15.4%).

**Table 3:** Patients’ demographic data

Parameter	Study population (***n*** = 59)		Survivors (n = 38)		Non-survivors (***n*** = 21)		***p***-value
	Mean	Std	mean	Std	mean	Std	
Age	76.9	6.3	75.3	5.6	79.6	6.8	**0.01**
Female gender [%]	51		55.3		42.9		0.37
Fracture location	6.9	2.0	6.9	2.1	6.9	1.8	0.99
Classification [OF]	4.1	0.7	4.1	0.7	4.1	0.7	0.93
ASA score	2.5	0.5	2.4	0.8	2.7	0.5	**0.02**
BMI [kg/m^2^]	27.0	4.3	27.2	4.3	26.6	4.5	0.63
Trauma mechanism	1.4	1.1	1.5	1.1	1.2	1.0	0.35
Duration surg [min]	142	46.2	134.8	40.8	154.8	53.8	0.16
Stabilized segments	4.3	1.4	4.2	1.6	4.4	1.0	0.46
Min. inv. Approach [%]	54.2		57.9		47.6		0.08
Adjacent injuries [%]	42.4		47.4		33.3		0.30
Thoracic cage inj [%]	22.0		28.9		9.5		0.06
Diabetes mellitus [%]	25.4		28.9		19.0		0.40
Renal insuffic. [%]	30.5		26.3		38.1		0.37
Heart insuffic. [%]	40.7		39.5		26.3		0.36
COPD [%]	13.6		7.9		23.8		**0.04**

*Std* Standard deviation; Fracture location: 3: thoracic vertebral body (TVB) 3: 4: TVB 4; 5: TVB 5; 6: TVB 6; 7: TVB 7; 8: TVB; 9: TVB 9; 10: TVB 10; Classification: 1: OF 1; 2: OF 2; 3: OF 3; 4: OF 4; 5: OF 5; ASA:; *BMI* Body mass index; Trauma mechanism: 0: not memorable; 1: low energy; 2: moderate energy; 3: high energy; surg: surgery; min: minutes; min. Inv. approach: Minimal invasive approach; inj: injury;insuffic.: insufficiency; COPD: Chronic obstructive pulmonary disease

A total of twelve sequential vertebral fractures were seen during the follow-up period (35.3%). There was a significant correlation between the occurrence of further vertebral fractures and high ODI scores (r = 0.476; *p* = 0.006) as well as high pain levels (r = 0.457; *p* = 0.009). Additionally, five complications were documented (8.5%) including wound healing disorders in three patients, a cement leakage with mild pulmonary embolism and one patient with pulmonary embolism who died during the hospital stay. Five revision surgeries were performed in five patients consisting of extension of the posterior stabilization in three patients because of adjacent fractures, removal of the implant in one patient due to implant-related complains and soft tissue revision due to a wound healing disorder in one patient. Besides, there was no clinical relevant cement leakage or implant loosening.

**Fig. 2:**
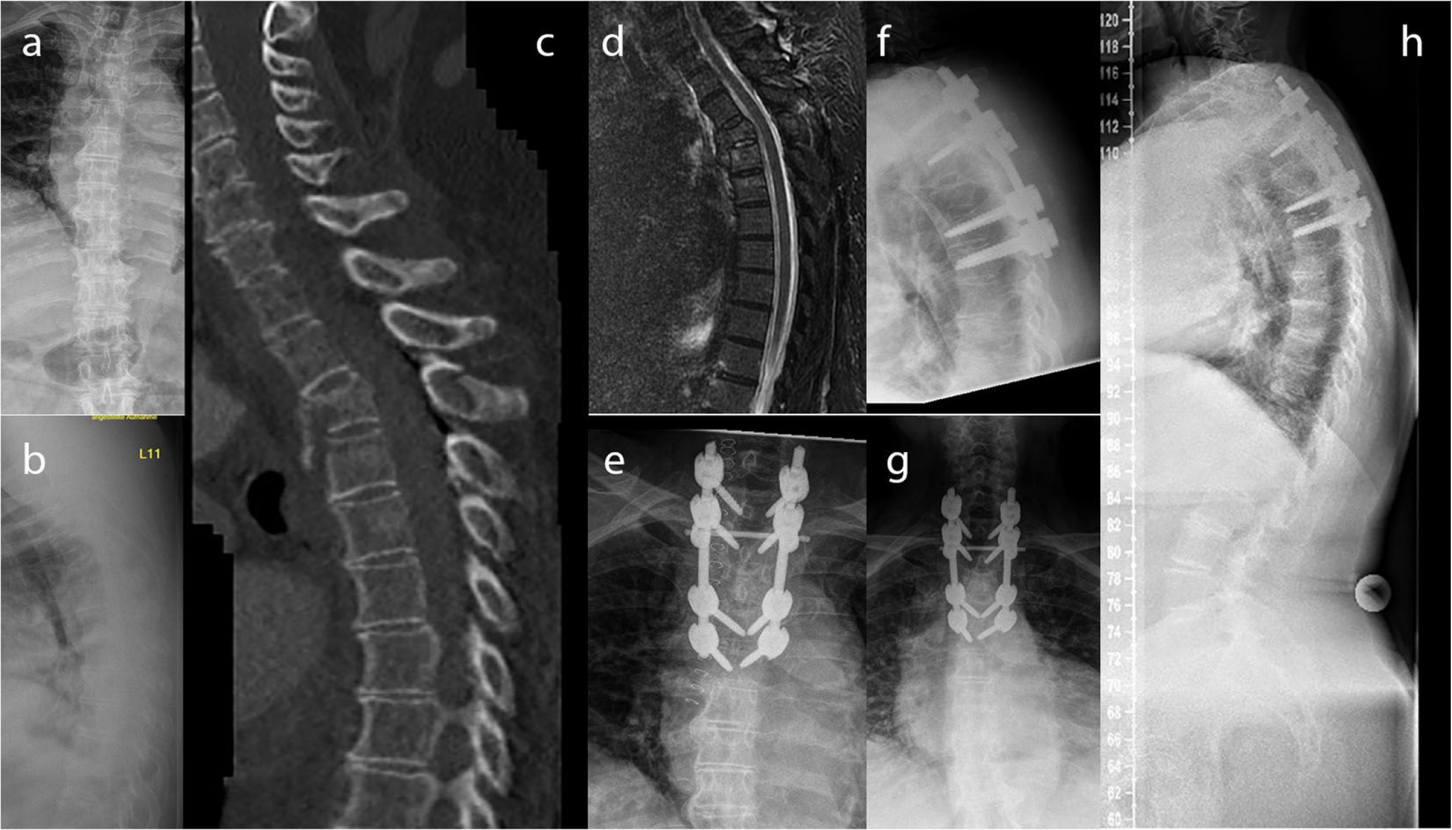
Seventy-eight-year female patient, who stumbled and fell down the stairs. A burst fracture of 3rd thoracic vertebral body with injury of the posterior column (OF 5) and an incomplete burst fracture of the 4th thoracic vertebral body was detected (**a-d**). Open posterior long-segmental stabilization was performed 3 days after the accident (**e, f**). At the latest follow-up after 26 months, the patient had only low pain levels without any pain medication (VAS: 2). No radiologic loss of reduction and compensated sagittal spinal alignment was visible (**g, h**)

**Fig. 3:**
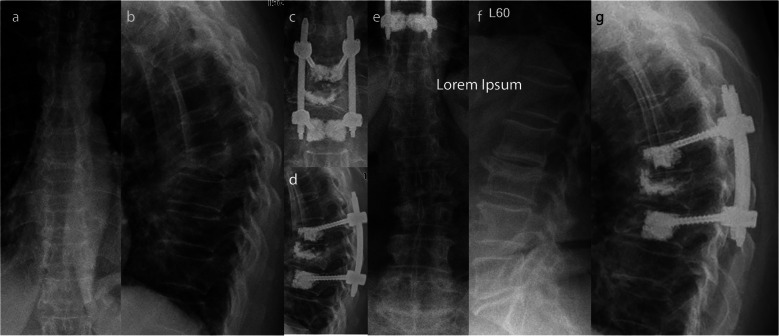
Sixty-eight-year female patient who suffered an acute incomplete burst fracture type OF 3 of the 8th thoracic vertebral body after falling while clearing snow. Surgery was indicated as result of the persistent immobilizing pain and the radiologic loss of reduction leading to a bisegmental kyphosis of 26° (**a, b**). A short segmental stabilization including cement augmentation of the fractured vertebral body was performed 7 days after the accident (**c, d**). At the further course an atraumatic subsequent fracture of the 3rd lumbar vertebral body occurred. At the latest follow-up after 69 months, the patient complained of permanent relevant pain (VAS: 6), moderate limitations (ODI: 45), and mild reduction loss as well as consolidated fracture 3rd lumbar body (**e, f**)

**Fig. 4:**
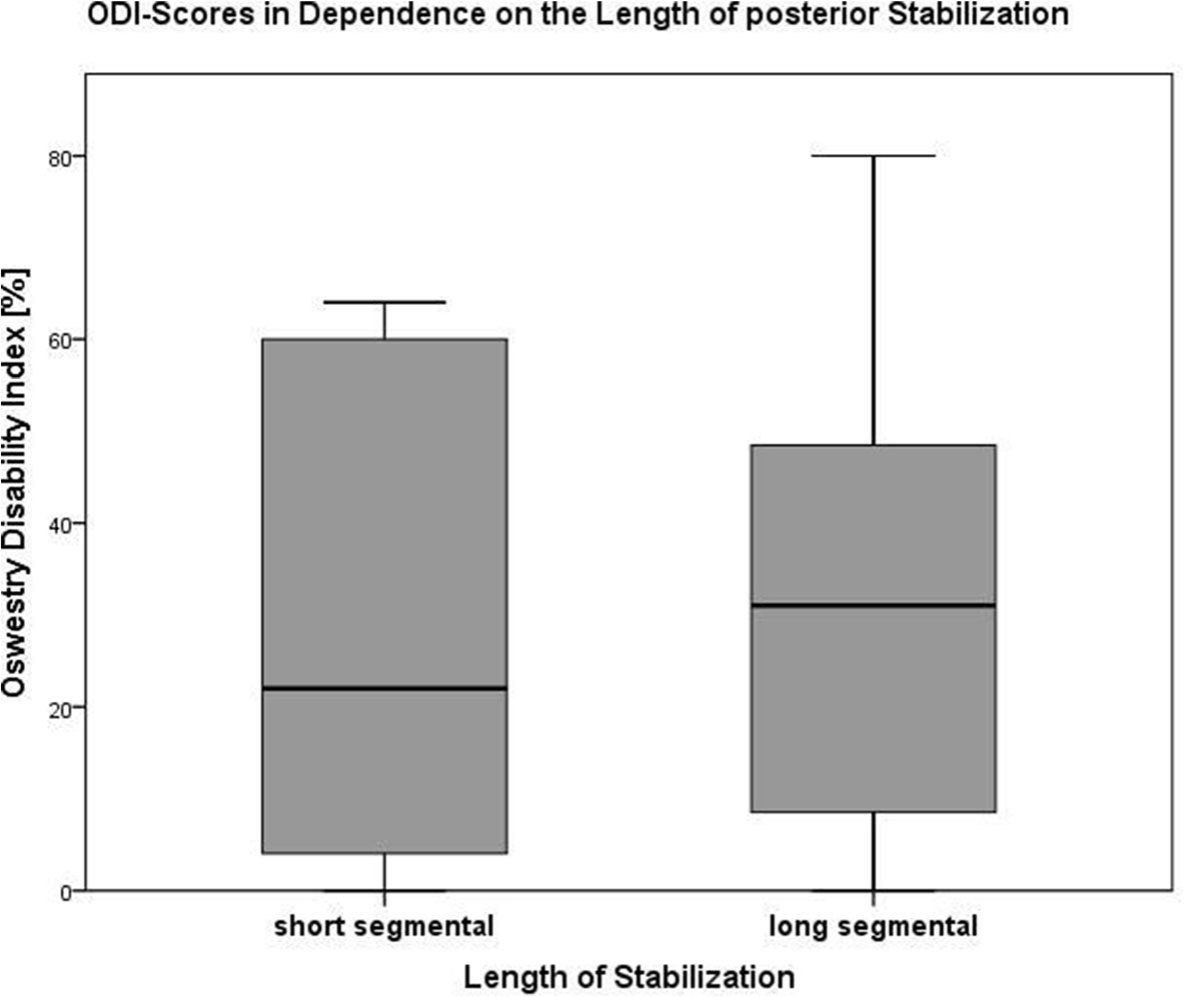
Box plot comparing the ODI scores between patients treated with short segmental stabilization (SSS) and long segmental stabilization (LSS) at the final follow-up examination after a mean of 5 years

**Fig. 5:**
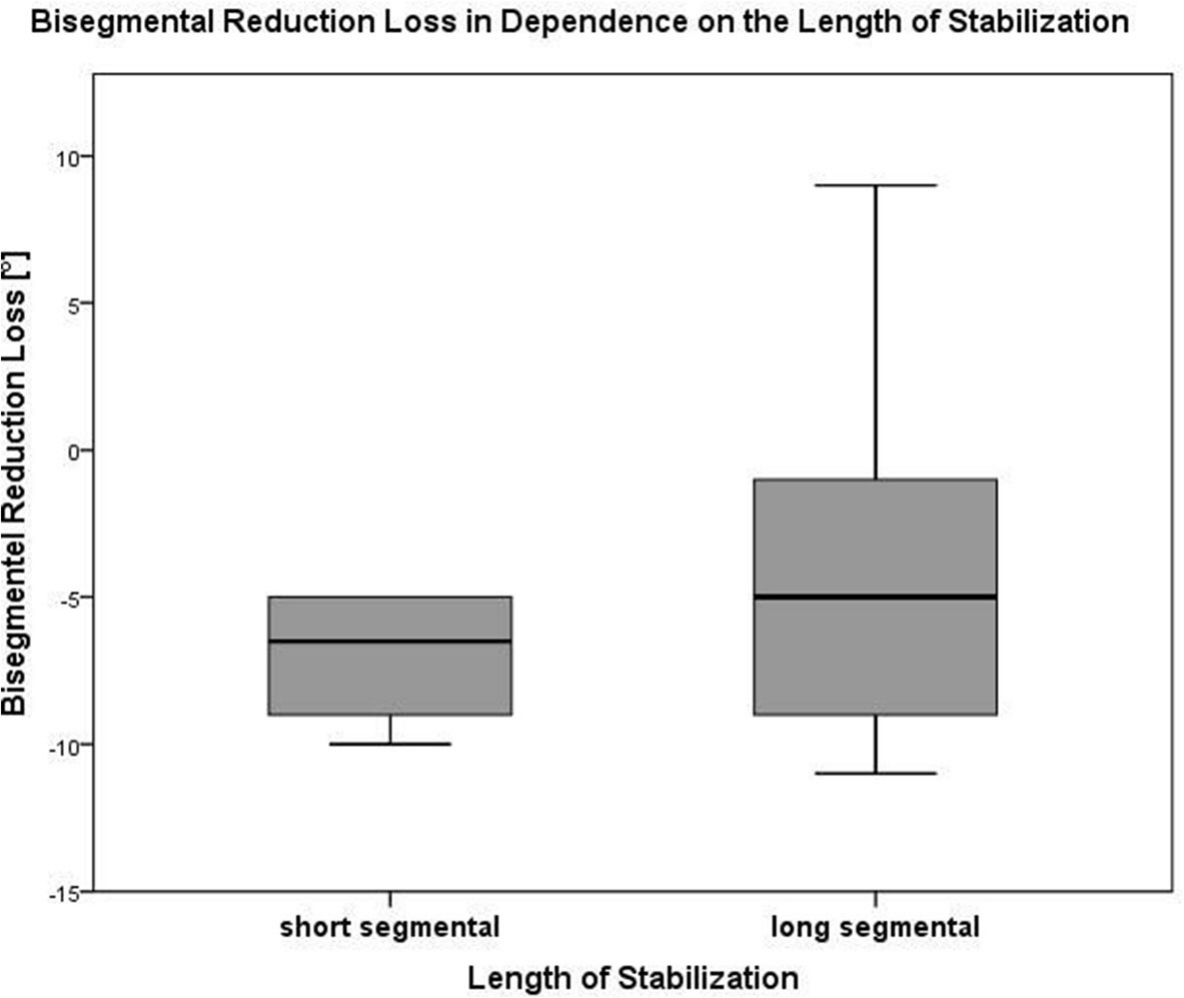
Box plot comparing the radiological bisegmental loss of reduction scores between patients treated with short segmental stabilization (SSS) and long segmental stabilization (LSS) at the final follow-up examination after a mean of 5 years

Twenty-nine of those 34 patients (85.3%), who were re-evaluated, were treated with LSS over a mean of 4.6 segments (range: 4–10) (Fig. 2). A minimal invasive approach was used in 18 patients (58.1%; open approach: *n* = 13; 41.9%). An additional kyphoplasty of the fractured vertebral body was performed in four patients (12.9%). The other five patients (14.7%) were treated bisegmentally (*n* = 3) or trisegmentally (*n* = 2 with one level above in one patient and one level below in another patient) (Table 4). Three of those were treated minimally invasive with kyphoplasty of the fractured vertebral body (Fig. 3). The primary outcome parameters are illustrated in Fig. 4 and Fig. 5. Altogether, sequential vertebral body fractures were seen significantly more often in patients with SSS than with LSS (80.0% versus 27.6%; *p* = 0.03). Thereby, the follow-up time was significant longer in the SSS patient group (83.4 months versus 54.3 months, *p* = 0.04). There was no significant association between concomitant injuries and any outcome scores nor with the mortality rate. However, there was a significant association between COPD and the mortality rate (*p* = 0.044) without any correlation with further risk factors. No further statistically significant differences were seen between both patient groups.

**Table 4:** Patients’ Outcomes in Dependence on Posterior Short- versus Long-Segmental Stabilization

Parameter	SSS (n = 5)		LSS (***n*** = 29)		p-value
	mean	range	mean	range	
ODI	30.0	0–54	31.5	0–80	0.92
PSC (SF-36)	33.6	17.2–51.8	33.6	17.5–62.1	1.0
Time-Up-and-Go Test [s]	9.0	7–11	11.3	5–38	0.94
Pain [VAS]	3.5	1–6	3.2	0–8	0.89
Reduction loss [°]	5.8	-1 - 10	4.1	0–13	0.45
Rel. med. Vertebral body height [%]	78.2	71–86	69.3	45–115	0.19
Thoracal kyphosise [°]	65.2	41–85	68.1	30–100	0.94
Sacral slope [°]	36.0	29–43	32.7	-1 - 62	0.95
Pelvic tilt [°]	20.5	19–22	21.4	7–41	0.95
Follow-up time [months]	83.4	50–111	54.3	18–103	**0.04**
Thoracic cage fractures [%]	0%		34.4%		**0.01**
Complication rate [%]	20%		13,8%		0.89
R. further vert. Fractures [%]	80%		27,6%		**0.03**

*SSS* Short segmental stabilization, *LSS* Long segmental stabilization, *ODI* Oswestry disability index, *PSC* Physical summary score, *NRS* Numeric rating score, *R.* Rate, *vert.* Vertebral

### Anti-osteoporotic therapy

Fifteen patients did not receive any anti-osteoporotic therapy (44.1%) despite the recommendation in the discharge report. Eleven patients had a non-specific anti-osteoporotic therapy (32.4%), whereas eight patients took bisphosphonates (23.5%).

## Discussion

The first main finding of this study was that unstable midthoracic fractures in geriatric patients can be caused by low to moderate trauma mechanisms with associated thoracic cage injuries. The second main finding is that the rates of adjacent thoracic cage injuries are high particularly in those with high energy trauma mechanisms. The third main finding is that the range of clinical and radiological results varied widely with a rather high mortality on the one side and minimal to moderate disabilities in 56.2% of the patients, rather low pain levels with 70.6% smaller or equal to 4 (VAS), and mainly low reduction losses of the survivors on the other side. However, the rates of further vertebral fractures was high, severe disabilities were seen in one third of the patients and high pain levels were seen in one quarter of the patients (≥ 6). Particularly, the occurrence of further vertebral fractures was associated with poor outcomes. Interestingly, patients treated with SSS had significantly higher rates of further vertebral fractures compared to those treated with LSS.

In literature, the majority of studies dealing with osteoporotic non-cervical fractures included the whole thoracolumbar spine [1, 4, 5, 19–24]. Thereby, the majority of fractures occurred at the thoracolumbar junction. In contrast, Ge et al. [25] and Ottardi C et al. [26] included osteoporotic thoracic fractures only. Ge et al. [25] included osteoporotic insufficiency fractures treated by cement augmentation, whereas Ottardi et al. [26] performed a finite element analysis of Th 10 fractures treated by vertebroplasty and kyphoplasty and demonstrated a positive correlation between the grade of vertebral body reduction and stress reduction on the vertebral body and concluded that in order to reduce the stress on the vertebral body a restoration of the physiological morphology is desirable.

In comparison to our results, Ge et al. [25] reported an inferior capacity of vertebral body restoration with a relative medial vertebral body height of 52%, even though the follow-up period was shorter (15 months) and the study group was younger (mean age: 70 years). Thereby, the final VAS score of 2.9 is in the range of our study group.

Generally, Wang et al. [5] reported a re-fracture or collaboration rate of the fractured or collapsed vertebral body of 38% 1 year after kyphoplasty. In contrast, we could persistently restore the vertebral morphology with a mean average medial vertebral body height of about 70% after LSS.

Gu et al. [27] reported a similar vertebral body height restoration of 77% after posterior stabilization in combination with augmentation of the fractured vertebral body at the thoracolumbar junction in patients with a comparable age. Additionally, the authors found a significantly better vertebral body height restoration, significantly lower Cobb angles, and a reduced number of adjacent fractures after additional posterior stabilization compared to kyphoplasty of the fractured vertebral body alone. Spiegl et al. [28] analyzed the structures which are responsible for the reduction loss after hybrid stabilization at the thoracolumbar junction and found the highest loss at the intervertebral disc adjacent to the fracture. Thus, the beneficial effect of a persistent restoration of the vertebral height on the sagittal alignment might be even more pronounced in the midthoracic spine based on the smaller intervertebral discs. In accordance to that, the mean loss of reduction seen in our patients was in the lower range in comparison to literature which ranged between 4.6**°** and 23° [6, 29–31].

Interestingly, there are only few studies dealing with osteoporotic fractures of the thoracolumbar spine and posterior stabilization [4, 6, 22, 23, 32, 33]. All of those studies included mainly fractures of the thoracolumbar junction. Studies exclusively dealing with posterior stabilizations of midthoracic fractures included mainly young patients with an average patient age ranging between 35 and 45 years [34–38].

Generally, the clinical outcome parameters are in the range of the results seen at the thoracolumbar spine of the elderly. Cheng et al. [2] reported mean ODI scores of 30.1% 2 years after kyphoplasty. Spiegl et al. [6] reported mean ODI scores of 29.9% 4 years after hybrid stabilization in elderly patients with comparable age.

Unfortunately, a high number of our patients did not receive a sufficient osteoporotic treatment despite a clear recommendation at the end of the hospital stay. Similarly, Aubry-Rozier et al. [39] reported that the percentage of patients having dual X-ray absorptiometry to diagnose osteoporosis was only 26% in patients treated by general practitioner, whereas this percentage was 72% if a fracture liaison service was used. Therefore, a simple recommendation for a further diagnostic work-up in the discharge report seems to be insufficient. In contrast, the diagnostic work-up should be initiated during the hospital stay or a liaison service needs to be started to optimize the osteoporotic therapy.

Most notably, SSS was significantly associated with higher rates of subsidence vertebral fractures compared to those patients treated LSS. However, no differences in the clinical outcome scores were seen between both subgroups. The missing differences in clinical outcome have to be put into perspective with the fact that patients treated with SSS did not suffer from any adjacent injuries of the thoracic cage. Surprisingly, concomitant injuries of the thoracic cage had no statistical impact on clinical outcome scores. In contrast, several studies reported negative effects of thoracic cage injuries such as serial rip fractures in the elderly [40, 41].

Generally, the indication for surgery has to be discussed critically in all patients [42]. Some of the patients might have comparable clinical outcomes without surgery or cement augmentation of the fractured vertebral body alone. Generally, we have seen the indication for an operative stabilization very strictly. Surgery was indicated in patients with unstable vertebral fracture and relevant destruction of the anterior column including complete burst fractures (OF 4) and type B and C injuries (OF 5) as well as a small number of patients (*n* = 6) with incomplete burst fractures and a relevant posterior wall involvement (OF 3) suffering from an immediate reduction loss of more than 5° after mobilization.

Altogether, this study offers several limitations. First of all, the retrospective study design has to be discussed critically. Particularly the decision making between SSS versus LSS as well as between minimal invasive versus open techniques was based on the surgeons’ experience and not the result of strict and objective criteria. Furthermore, comparison groups such as patients treated with cement augmentation alone or non-operative treated patients are missing. However, we believe there is a sufficient evidence of posterior stabilization in unstable thoracolumbar fractures that justifies our strategy. Furthermore, patients with neurologic deficit were excluded. The reason for excluding these patients was the surprisingly low number of patients with neurologic deficits who were treated in our clinic during the study period (*n* = 5). Additionally, the mortality rate of our study was high. Excluding those patients, the follow-up rate was close to 90% which is extraordinarily high considering the high patient age and the long follow-up period. Besides, no sufficient diagnosis of osteoporosis was performed and no anti-osteoporotic therapy was started in the majority of the patients despite the clear recommendation for it in the discharge report. The low number of patients who received sufficient antiosteoporotic therapy might be partially responsible for the high rate of sequential vertebral fractures and might negatively affect clinical outcomes [43].

Altogether, the specific study group with unstable fractures of the midthoracic spine in the elderly with a very high follow-up rate of the surviving patient and the long follow-up time is a strength of this study. Based on these results, the authors changed their diagnostic strategy by performing a CT examination including the entire thoracic cage in all elderly patients suffering from a midthoracic fracture in order to not miss frequent concomitant thoracic injuries. Additionally, we indicate LSS including two vertebral bodies above and below the fracture and use a minimal invasive approach if possible. Furthermore, antiosteoporotic diagnostic will be started initially during the inpatient stay and a fracture liaison system was initiated.

## Conclusion

Unstable fractures of the midthoracic spine are associated with high rates of thoracic cage injuries. The mortality rate was rather high. The majority of the survivors had minimal to moderate disabilities. Thereby, patients treated with LSS had a significantly lower rate of sequential vertebral body fractures during follow-up.

## Data Availability

The datasets from the study are available from the corresponding author on reasonable request.
